# Efficacy of a 2‐MNG‐Containing Depigmenting Serum in the Treatment of Post‐Inflammatory Hyperpigmentation

**DOI:** 10.1111/jocd.16735

**Published:** 2024-12-26

**Authors:** Ann Laure Demessant‐Flavigny, Gitanjali Petkar, Dylan Jodun, Guénaëlle Le Dantec, Caroline Le Floc'h, Delphine Kerob

**Affiliations:** ^1^ La Roche Posay Laboratoire Dermatologique Levallois‐Perret France; ^2^ Centre International développement Pharmaceutique, CIDP Phoenix Mauritius

**Keywords:** depigmenting, efficacy, hyperpigmentation, PIHP, skincare

## Abstract

**Background:**

Post‐inflammatory hyperpigmentation (PIHP) predominantly affects patients with melanin‐rich skin, significantly impacting them psychosocially due to more frequent and severe pigmentary changes. In this study, the efficacy of a novel depigmenting agent 2‐mercaptonicotinoyl glycine (Melasyl) in a dermocosmetic (DC) serum formulation is assessed as a stand‐alone treatment of PIHP without sunscreen.

**Materials and Methods:**

Thirty‐two Mauritian subjects aged 18–50 years of phototype IV–VI presenting mild acne (GEA2) and moderate to severe PIHP (PAHPI > 10) participated in this study. Subjects applied the DC serum twice a day on the whole face for 3 months. Efficacy was assessed through PAHPI score, mean darkness of lesions (0–8 scores), and colorimetric measurements at D0, D14, D28, D56, and D84. Self‐perceived efficacy, tolerability, stigmatization, cosmeticity, and satisfaction were also gathered.

**Results:**

A significant decrease of 15.8% (*p* < 0.05) in PAHPI score was reported at D84. The PAHPI score showed marked changes (*p* < 0.05) in pigmentation intensity at D56. Visible significant changes in mean darkness were observed from D28, with a 25.1% decrease at D84. A significant brightening effect was observed in both the spots and adjacent areas, with their mean ITA values increasingly converging toward the ITA of the non‐treated, non‐exposed zone. This effect was more pronounced in the spots, which showed a significant increase from −34.1 to −14.2 by D14. Instrumental measurements revealed a 60% reduction in spot color intensity at D84 compared to the adjacent area Delta *E* (*p* < 0.05), significant from D14. Subjects reported self‐perceived improvements in appearance and well‐being that matched clinical results, enhancing their quality of life and satisfaction. The product was reported as very well tolerated by the subjects after 84 days of usage.

**Conclusion:**

This study demonstrated the efficacy and tolerability of the serum in the treatment of post acne PIHP. Good clinical results are confirmed by the objective measurement of the ITA and Delta *E*. Cosmeticity was excellent and will help observance in real life. The reduction of the stigmatization score illustrates the impact of PIHP and its improvement over time. These results can be considered highly encouraging with the new serum, as in real life, its use in combination with a UVA/visible light filtration sunscreen would probably increase patients benefit.

## 1 Introduction

Hyperpigmentation relates to the darkening on the skin's natural tone, typically resulting from an excessive deposition of melanin (hypermelanosis) in either the dermis or epidermis [1]. Acne (acne vulgaris) is a prevalent chronic inflammatory skin condition of the pilosebaceous unit predominantly on the face [2]. Alongside acne, individuals with melanin‐rich skin (Fitzpatrick phototypes IV–VI) often exhibit post‐inflammatory hyperpigmentation (PIH) or PIHP [3, 4, 5]. These can last at least a year and up to 5 years as the severity of acne lesion increases, qualifying PIHP as more problematic for patients than acne itself [3]. The location and the severity of skin discoloration can substantially impact their quality of life (QOL) leading them to endure significant psychological distress [5, 6].

The existing body of literature concerning PIHP associated with acne is notably limited, especially with regard to its pathogenesis and strategies for treatment [3, 5, 6]. Moreover, acne‐induced PIHP cases require prompt treatment [7] and can take more time to improve the point of restoration to normal skin pigmentation thus further impairing the well‐being of patients seeking dermatological help [5, 8].

Currently, topical formulations of conventional agents remain the primary option of front‐line treatment for the post‐acne PIHP. The most commonly used agents are hydroquinone, arbutin, kojic acid and niacinamide. Hydroquinone and its derivative arbutin act by suppressing melanin synthesis [7, 9, 10]. They prevent the conversion of l‐3,4‐dihydroxyphenylalanine (l‐DOPA) to melanin by inhibiting tyrosinase [11, 12]. Niacinamide helps improve the skin barrier function [13, 14, 15]. Suncare products help prevent neo‐melanogenesis and further darkening existing spots [16, 17]. Non‐topical treatments usually consist of microdermabrasion and non‐ablative laser interventions [16, 17, 18].

However, these common depigmenting agents have been shown to potentially trigger skin irritation in patients dealing with dermal sensitivity issues. For instance, irritation caused by hydroquinone at 4% can exacerbate PIHP, particularly in darker skin types and its prolonged use can lead to ochronosis, a condition especially prevalent in individuals with melanin‐rich skin [19].

Hyperpigmentation is a complex phenomenon and thus requires a holistic treatment strategy that addresses all these mechanisms. Treatments should, for instance, encompass exfoliating actions to target the epidermis, fortify the skin barrier to prevent melanin leakage from the basal membrane to the dermis, inhibit melanogenesis, incorporate anti‐inflammatory and antioxidant actions [8]. Furthermore, reinforcing the dermal component is essential for effectively managing and reducing hyperpigmentation.

Recently, the novel molecule, 2‐mercaptonicotinoyl glycine (2‐MNG/Melasyl) was identified and characterized in vitro and ex vivo where it exhibited a high performance thanks to two additive modes of action [20]. Unlike other conventional agents, 2‐MNG has an innovative mode of action by conjugating with melanin precursors to inhibit their conversion to eumelanin and pheomelanin pigments. Furthermore, 2‐MNG was demonstrated to deprive melanocytes of other highly reactive melanin precursors such as dopaquinone (DOPAQ) thus reducing its chemical reactivity [20]. In the clinical setting, 2‐MNG yielded promising results in melanin‐rich skin (phototypes III–V) with a very good safety profile and no tolerability issues in a healthy panel of volunteers who were exposed for four consecutive days to a daily dose of 0.5 individual minimum erythemal dose (MEDi) of UV daylight (UVDL) produced by a solar simulator [21]. In this study, 2‐MNG prevented immediate darkening and inhibited neo‐melanogenesis. Finally, 2‐MNG had a broad spectrum of action in melanin‐rich skin, which attest to its versatility and inclusivity [20, 21].

While 2‐MNG is an efficient and well‐tolerated candidate for safe skin tone and hyperpigmentation management, it is important to understand its facial anti‐pigmenting and depigmenting activity with respect to moderate to severe facial PIHP due to acne in a considerable time period and demonstrate its ability to improve global QOL.

Here, the effects of 2‐MNG were evaluated in a single site, full face and full arm study, with the DC serum as test product. This clinical study focused on healthy individuals of Fitzpatrick phototypes IV–VI, with moderate to severe PIHP associated with mild acne following a 12‐week product application period. This study highlights the ability of a 2‐MNG‐containing formula to improve the skin's complexion without the use of suncare products in an inclusive approach which involves a close follow‐up and assessment of the cases by clinical investigators, instrumental measurements and considers the evolution of perceived PIHP by the participants.

## 2 Materials and Methods

### 2.1 Study Overview

This was an open‐label study conducted in a single investigational center in Mauritius between April and August 2023 for 12 weeks. The study design is described in Table 1.

**TABLE 1:** Study flow chart.

	Visit to centres				
	D0	D14	D28	D56	D84
Informed consent	X	—	—	—	—
Verification and confirmation of inclusion/exclusion criteria	X	—	—	—	—
Acclimatization	X	X	X	X	X
Demographic data	X	—	—	—	—
Skin examination	X	X	X	X	X
Clinical scoring	X	X	X	X	X
Examiner counting lesions	X	X	X	X	X
Colorface imaging	X	X	X	X	X
Subject global assessment (SGA) by subjects	—	X	X	X	X
Colorimeter measurement on dark spot (×3)/adjacent skin/unexposed	X	X	X	X	X
Subjective assessment of global tolerance	—	—	—	—	X
Product dispensation and application at center	X	—	—	—	—
Subject Questionnaire: Stigmatization questionnaires	X	X	X	X	X
Subject Questionnaire: Cosmetic	—	—	—	—	X
End of study/compliance check	—	—	—	—	X

### 2.2 Ethical Considerations

The study (Trial Registration Number: ECC‐COS‐083) was conducted in accordance with the guidelines of the Declaration of Helsinki and OECD good clinical practices (GCP). The study was conducted within the national guidelines of Mauritius and obtained a favorable opinion by Life Together Ltd‐Ethics Committee (IEC) on March 8, 2023. Informed written consent was obtained from all subjects after explaining the study procedures, restrictions, potential risks, and benefits, their right to withdraw at any time, and the notification procedures for adverse events.

### 2.3 Subjects

A group of 32 healthy subjects aged between 18 and 46 years was enrolled to participate in the study. The subjects were of skin phototypes IV–VI (Fitzpatrick scale) and present mild acne on the face (Based on the Global Acne Evaluation, GEA = 2, and < 9 inflammatory lesions in total), and moderate to severe PIHP with a Post Acne Hyperpigmentation Index (PAHPI) score > 10. Demographic distribution of the subjects was detailed in Table 2.

**TABLE 2:** Subject demographic distribution.

Inclusion population	*n* = 32
Male/Female (%)	1 (3%)/31 (97%)
Age (mean, min–max)	31 years old (18–46 years old)
Phototypes (%)	IV: 16 (50%)
	V: 14 (44%)
	VI: 2 (6%)
Ethnicity (%)	African: 1 (3%)
	Indian: 5 (16%)
	Mixed‐race: 26 (81%)
PAHPI at baseline	13.9

### 2.4 Test‐Application and Visits

The tested product was a serum containing 0.5% 2‐MNG (Melasyl), 10% Niacinamide, *Cystoseira tamariscifolia* extract, LHA, Carnosine, Retinyl Palmitate and Dipotassium Glycyrrhizate (MelaB3 serum, La Roche‐Posay Laboratoire Dermatologique, France), hereafter DC. All patients in the study were instructed to apply the DC serum on the whole face twice daily in the morning and the evening from Day 0 (D0) to Day 84 (D84) and advised to protect themselves from sun exposure. During the 12‐week study period, the subjects visited the center at D0, D14, D28, D56, and D84 for evaluations.

### 2.5 Clinical Evaluations

On D0, D14, D28, D56, and D84, the dermatologist performed clinical evaluation grading of facial characteristics. A dermatologist examined the subjects and attributed them with clinical scores using PAHPI score (based on the number of lesions, their size, and intensity), mean darkness of PIHP lesions (from 0, normal to 8, very distinctive), GEA (from 0, clear to 5, very severe), and acne lesions counts (non‐inflammatory, inflammatory, and total count).

### 2.6 Instrumental Evaluations

Using a spectrocolorimeter CM700d (Courage + Khazaka electronic GmbH, Koln, Germany), spectrocolorimetric measurements (L*a*b*; ITA°, Delta *E*) were taken at D0, D14, D28, D56, and D84. Three PIHP lesions (denoted as spots) and three corresponding adjacent zone (denoted as adjacent) were selected on the face, all of which received treatment and were susceptible to sun exposure. Colorimetric measurements were also taken as a control underneath the arm area which remained as untreated and unexposed zones (NTNEZ).

Standardized pictures of the treated areas were taken for illustrative purposes using ColorFace Imaging system (Newtone Technologies, Lyon, France) consisting of a DSLR camera equipped with a high‐resolution imager (24 M pixels). The ColorFace system captured front, left, and right views in all modalities: Cross‐polarized (CP), Standard 60 (STD 60), Standard 45 (STD 45), No filters images, Parallel‐polarized (PP), illustration (ILLUS), and UV. Renaming of the images was standardized according to the software program.

### 2.7 Self‐Assessment Questionnaires: Efficacy, Quality of Life, Cosmeticity, and Satisfaction

The subjects contributed to the evaluations via self‐assessment questionnaires at D0, D14, D28, D56, and D84 regarding subject global assessment of PIHP (from −1, worsened to 4, totally cleared), local tolerance, stigmatization (PUSH‐D questionnaire gathering eight questions on the felt stigma and nine questions on the enacted stigma), cosmeticity, and efficacy and satisfaction.

### 2.8 Self‐Assessment Questionnaires: Stigmatization Assessment

At Visit D0, D14, D28, D56, & D84 subjects completed, at the clinical site, a subject questionnaire designed for subjects with visible skin conditions (PUSH‐D), based on their feelings or perceptions, to assess felt (eight questions) and enacted (nine questions) stigmatization.

### 2.9 Statistical Analysis

Qualitative variables were summarized using counts and percentages, while numerical ones, in the form of central tendencies such as the mean and median and dispersion (SD). The change from baseline was assessed at all timepoints using the paired samples *t*‐test or the Wilcoxon signed rank test, depending on the normality of the difference data, all at 5% level of significance (two‐sided). Normality was tested at 1% significance level of Shapiro–Wilk test. No multiplicity adjustments were performed. Graphical illustrations indicate the mean ± 95% CI. All inferential analyses were run in IBM SPSS Statistics (Version 19.0).

## 3 Results

### 3.1 Global PAHPI Assessment

**FIGURE 1:**
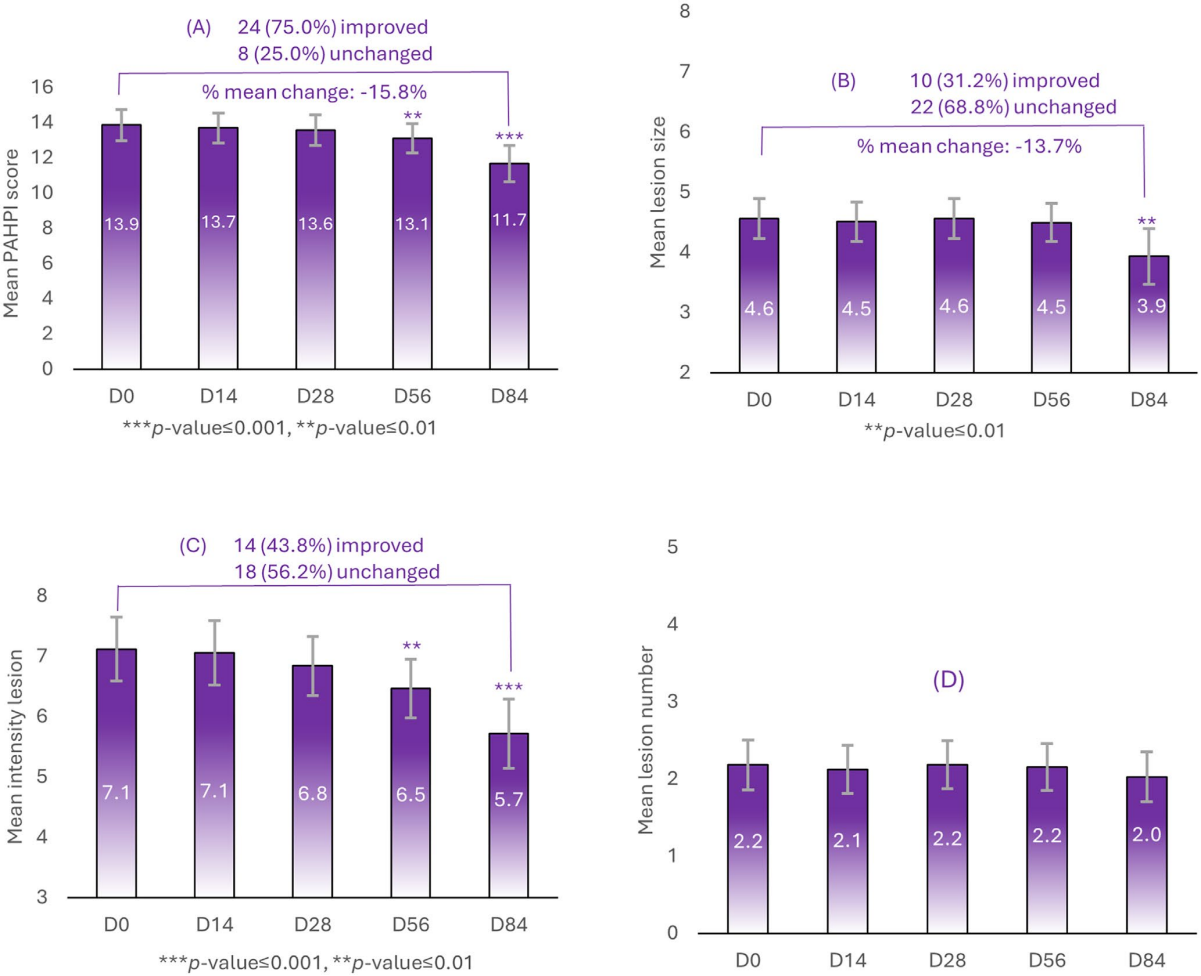
Global PAHPI (A) score reported in *n* = 34 subjects between D0, D14, D28, D56, and D84. (B) Percentage of subjects (*n* = 32) with improvements in PAHPI scores (*n* = 24) (denoted in blue) versus subjects whose clinical signs remained unchanged (*n* = 8) (denoted in gray). Changes in individual PAHPI parameters (C) lesion size, (D) intensity of lesions, (E) lesion number. ** a significancy of *p* < 0.01 and *** a significancy of *p < *.001.

A mean change of 15.8% in PAHPI score at D84 compared to the baseline was reported globally (*p* < 0.05). An improvement in PAHPI score of 75.0% was also reported in subjects (*n* = 24) as compared to the remaining 25.0% of subjects (*n* = 8) (Figure 1A).

When individual parameters of PAHPI were assessed, a significant mean change of 13.7% in lesion size from D0 to D84 was observed where a 31.2% (*n* = 10) improvement was noted and 68.8% (*n* = 22) remained unchanged (Figure 1B). A significant decrease in the intensity of lesions was, however, noted as early at D56 when compared to the baseline, D0 where *n* = 14 (43.8%) showed improvement and *n* = 18 (56.2%) remained unchanged after D84 (Figure 1C). The number of lesions remained relatively unchanged between D0 and D84 (Figure 1D).

### 3.2 Mean Darkness Clinical Evaluation

**FIGURE 2:**
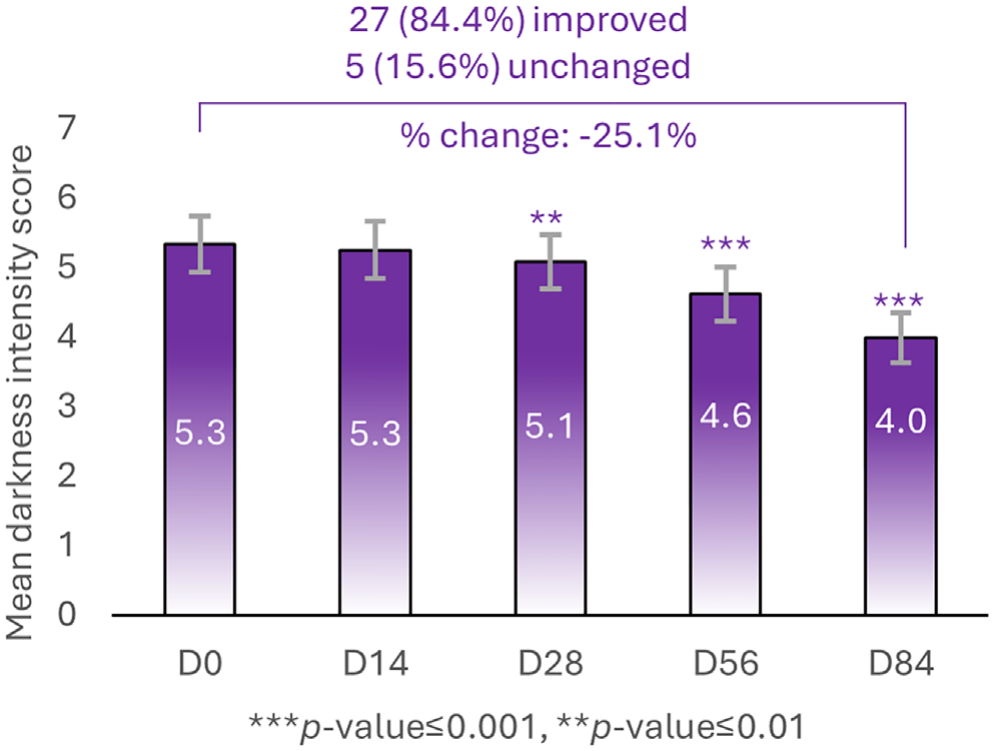
Clinical evaluations of mean darkness. Changes in mean darkness values at different timepoints (D0, D14, D28, D56, and D84). The Percentage of subjects (*n* = 32) with improvements in darkness scorewere 84.4% (n=27)(versus 15.6% subjects (n=5) whose clinical signs remained unchanged. ** a significancy of *p* < 0.01 and *** a significancy of *p* < 0.001.

In 84.4% of subjects (*n* = 27), the mean darkness clinical evaluation (where 0 = normal and 8 = severe/very distinctive) showed a significant improvement at D28 with a mean darkness score of 5.1 (*p* < 0.05), 4.6 (*p* < 0.05) at D56 and 4.0 (*p* < 0.05) at D84 compared to the baseline, D0 (Figure 2). At D84, the mean change was 25.5% and the mean value for the remaining 15.6% of subjects (*n* = 5) remained unchanged (Figure 2).

### 3.3 Global Assessment Evaluations (GEA) for Acne Parameters

As part of the GEA, the total lesion count had a gradual decrease as early as D14. The mean value at baseline was 26.9 with a significant decrease noted as from D28 with a mean value 23.3 (*p* < 0.05). Further significant decrease was noted at D56 with a mean value 19.2 (*p* < 0.05) and D84 showing the highest significant decrease of 43.8% (*p* < 0.05) compared to the baseline, D0 with a mean value of 15.1.

### 3.4 Instrumental Evaluation of Pigmental Changes Measured by Chromameter

**FIGURE 3:**
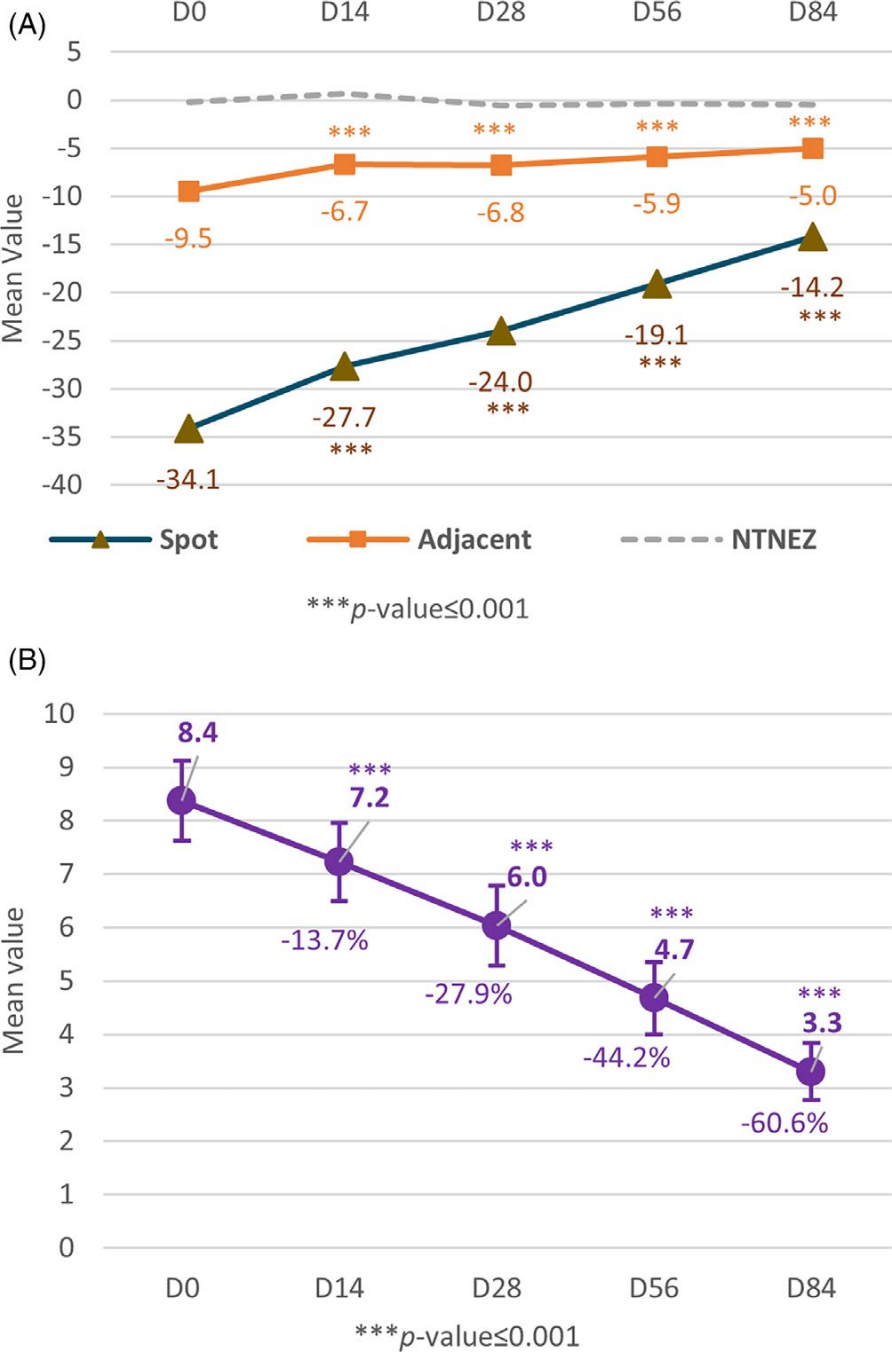
Spectro‐colorimetric measurements at D0, D14, D28, D56, and D84 of PIHP lesions (A) ITA values of PIHP scores on three different zones (spot, adjacent, and NTNEZ) and (B) delta *E* values. *** a significancy of *p* < 0.001.

A brightening action is observed in both the spot and the adjacent area as their respective mean ITA° values increase and converge toward the ITA° of the NTNEZ (Figure 3A). This increase was significant but less pronounced in adjacent zones compared to the spots which were more significantly noticeable as soon as at D14, where the mean ITA° value increased from −34.1 to −14.2 on the spot zone (*p* < 0.05), and −9.5 to −5.0 on the adjacent area (*p* < 0.05). This is also represented as a 60% reduction in contrast to Delta *E*, that is, the difference in color intensity between spot and adjacent area, at D84. This decrease was significant as at D14 (*p* < 0.05) (Figure 3B).

### 3.5 Illustrative Cases

**FIGURE 4:**
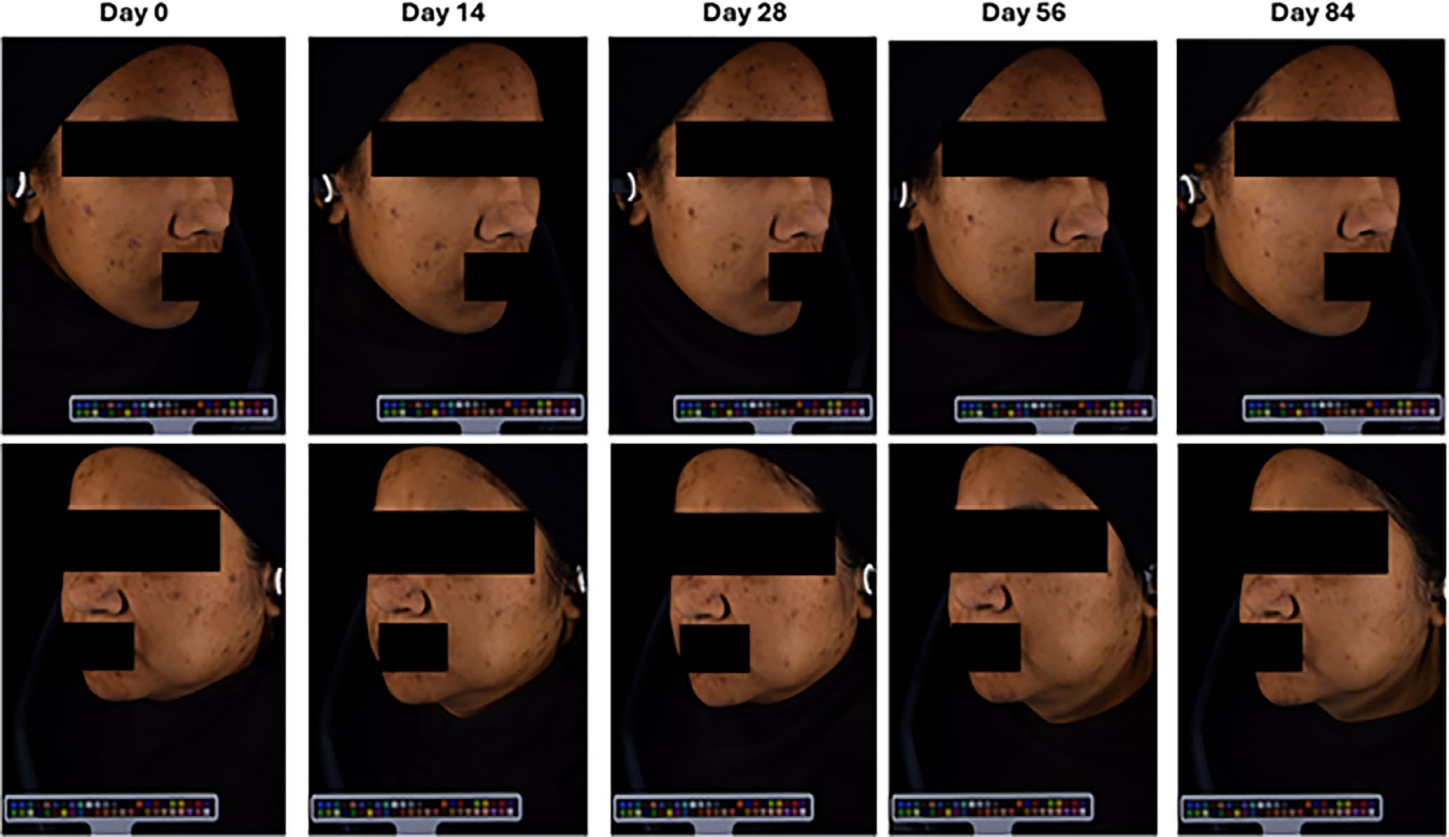
Photographs of the face of an anonymized subject after 84 days of treatment. Ambient lighting was maintained throughout the study. From left to right, panels show the face at D0, D14, D28, D56, and D84, respectively.

The evolution of two patients after treatment with the serum was followed from D0 to D84. From Figure 4, improvement in PIHP‐related symptoms was observed.

### 3.6 Subject Global Assessment of Skin Improvement (SGA)

**FIGURE 5:**
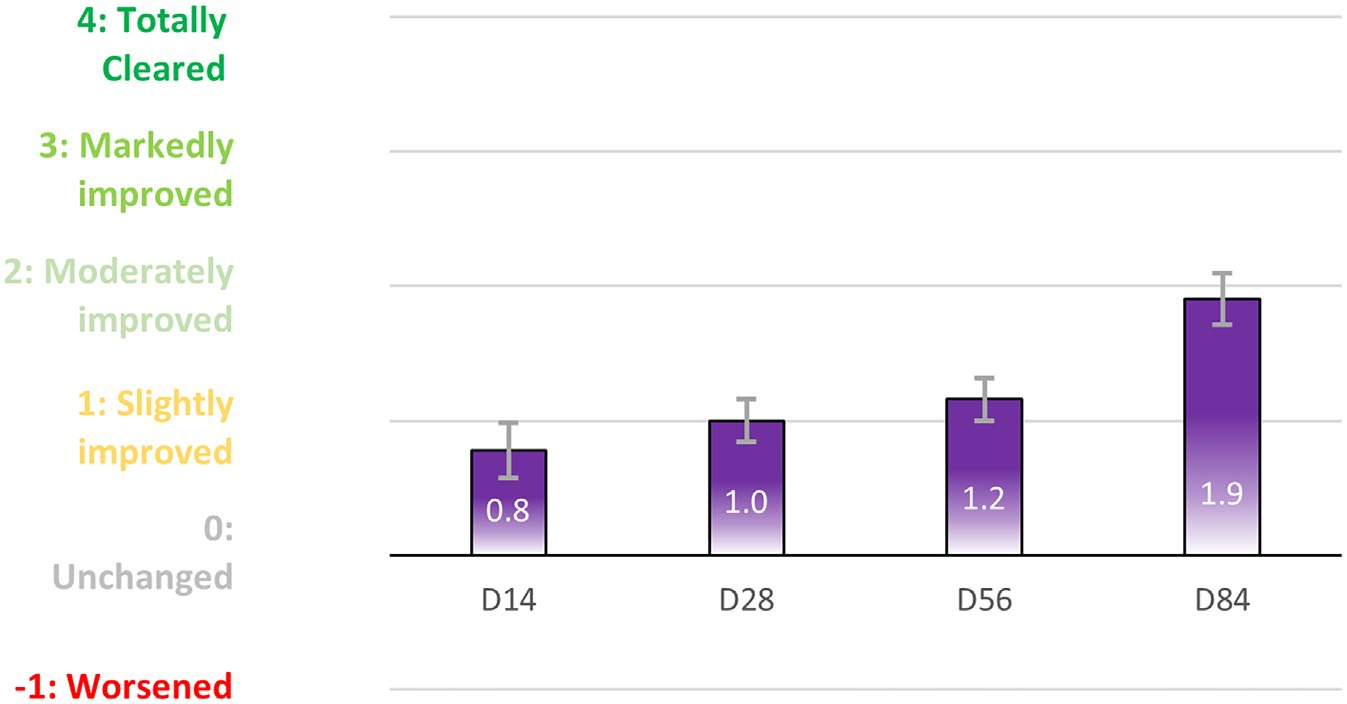
Subjects responded about the improvement of their skin by self‐evaluation at D14, D28, D56, and D84 visits. Average score was recorded for phototype IV–VI. Treatment comparisons was not significant.

Subjects pointed to a moderate improvement of self‐perceived PIHP, from an average score of 0.8 (slight improvement) at D14 to an average score of 2.0 (moderately improved) at D84 (Figure 5).

### 3.7 Stigmatization Global Scoring

**FIGURE 6:**
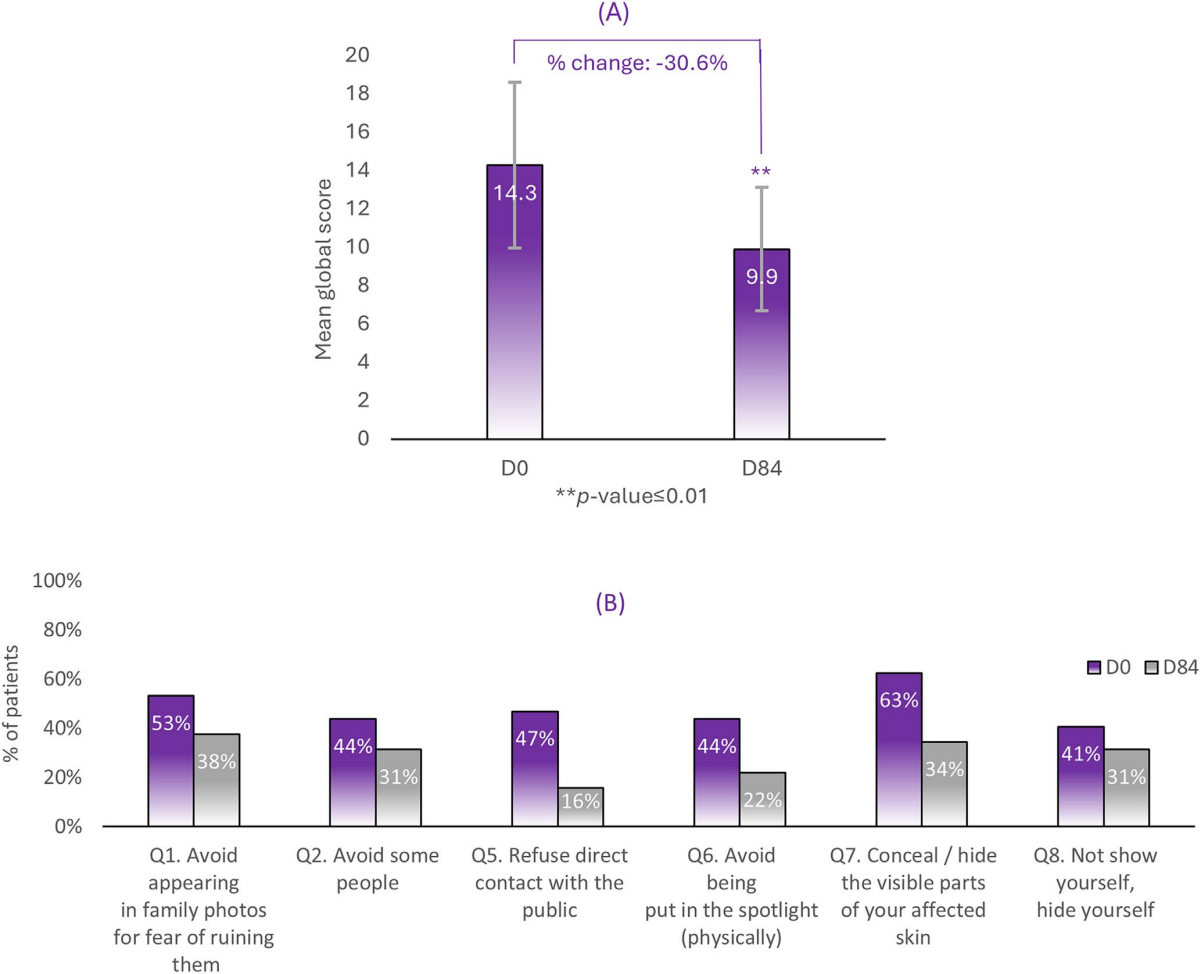
At Visit D0 and D84, subjects completed a questionnaire based on their feelings or perceptions, to assess stigmatization. (A) The global score. (B) The stigma felt by subjects. (C) The enacted score refers to the stigma perceived by subjects. ** a significancy of *p* < 0.01.

Overall, the stigmatization questionnaires showed a 30.6% significant decrease between the global stigmatization scores from D0 to D84 (Figure 6A). Notably, 63.0% of subjects who hid the visible part of affected skin at D0 fell to 34.0% at D84. Moreover, 47.0% of subjects who refused direct contact with the public at D0 dropped to 16.0% at D84. Lastly, 44.0% of subjects who avoided being in the spotlight decreased to 22.0% at D84 compared to D0 (Figure 6B).

### 3.8 Product Cosmeticity Assessment and Acceptability

More than 85.0% of the subjects had a favorable opinion of the product with regard to its efficacy and cosmetic attributes (Tables 3 and 4). Accordingly, all subjects found the serum to perform better than their usual anti‐dark spot treatment and 97.0% of them prefer the product to their current product (Table 5). Furthermore, the product formulation tested by the subjects can be considered very well tolerated after 84 days of twice daily use on the face.

**TABLE 3:** Subjects (*n* = 32) provided their experience by answering a cosmetic evaluation questionnaire at D84 where each statement represented their appreciation of the product.

Cosmetic items	Agree/somewhat agree
The product has a pleasant texture	100%
2 The product has a fresh texture	100%
3 The product leaves a comfortable sensation on skin	100%
4 The product instantly illuminates skin	97%
5 The product does not cause any reaction (redness, stinging, tingling…)	88%
6 The texture is light weight	100%
7 The product has a pleasant smell	97%
8 The product has an efficacious smell	97%
9 The product is easy to apply, to spread	100%
10 The product does not go to noodles on my skin	100%
11 The product is gentle for the skin	100%
12 The product doesn't leave the skin oily, greasy	97%
13 The product does not leave the skin shiny	88%
14 The product does not feel sticky on the skin	91%
15 The product is quickly absorbed	94%
16 The product allows the skin to breathe	97%
17 The product doesn't leave white marks on skin	94%
18 The product does not leave marks on clothes/other fabrics	91%
19 The product is suitable for my skin type	100%
20 Is an optimal make‐up base	88%

**TABLE 4:** Subjects (*n* = 32) provided their experience by answering an efficacy evaluation questionnaire at D84 where each statement represented their appreciation of the product.

Efficacy item	Agree/somewhat agree
Skin is brighter	97%
2 Skin tone looks more even	100%
3 Skin seems fairer	100%
4 Dark spots size looks reduced	100%
5 Number of dark spots looks reduced	100%
6 Color of dark spots looks lighter	100%
7 Persistent dark spots look less visible	94%
8 The most persistent dark spots look less visible	97%
9 Oldest dark spots look less visible	100%
10 Dark spot area looks less visible	100%
11 Imperfections look reduced/minimized	100%
12 Skin complexion appears fresher	100%
13 Skin is more luminous/radiant	100%
14 Product provides a healthy glow	100%
15 My skin looks younger	91%
16 Wrinkles appear reduced/minimized	76%
17 Skin texture appears refined	94%
18 Visible pores look reduced/minimized	94%
19 Fine lines appear reduced/minimized	88%
20 Skin is softer	97%
21 Skin is smoother	100%
22 Skin is renewed	97%
23 Skin is hydrated	94%
24 Skin is nourished	97%
25 Skin feels comfortable	100%
26 Skin feels soothed	97%

**TABLE 5:** Subjects (*n* = 32) provided their satisfaction on the product after 84 days of usage.

I find this product more efficacious than my usual anti‐dark spot	100%
2 I prefer this product than my current product	97%

## 4 Discussion

Acne‐induced PIHP is a prevalent complication of acne, particularly affecting individuals with darker skin phototypes. This condition arises from the inflammatory response to acne lesions, leading to an excessive deposition of melanin in the affected area. PIHP can significantly impact the QOL (e.g., having difficulty socializing, feeling self‐conscious, isolated or embarrassed) of individuals with darker skin, causing psychological distress and stigmatization due to its visible and persistent nature [5, 18, 21]. The psychological burden of PIHP underscores the importance of effective treatment strategies to alleviate both physical and emotional well‐being [5, 21].

In addressing acne‐induced PIHP, the study introduces 2‐MNG (Melasyl) as a prospective candidate. It stands out in skin pigmentation management for its unique dual mode of action, acting as a trapper directly targeting melanin precursors to inhibit melanin synthesis while reducing melanocyte reactivity [20]. Clinical studies demonstrate its efficacy in melanin‐rich skin types in a UV‐induced pigmentation model without adverse effects, highlighting its versatility and good safety profile [21]. For instance, a Bayesian network meta‐analysis confirmed its effectiveness by comparing its superior action against hyperpigmentation against multiple other established treatments [22]. Therefore, 2‐MNG provides a favorable solution to achieve a balanced and radiant skin tone with minimal risk in patients with PIHP spots. The DC facial serum contains 2‐MNG at 0.5% as the key ingredient to decrease both eumelanin and phaeomelanin synthesis in skin. Additionally, other active ingredients complement the formula to answer the complex pathophysiology of PIHP [5]: LHA (lipohydroxy acid) and retinyl palmitate were instrumental in promoting exfoliation and strengthening the epidermal junction, thus improving skin texture and barrier function [23, 24]. Carnosine acted as a potent antioxidant, protecting the skin from oxidative stress [25]. Niacinamide and K2G exhibited notable anti‐inflammatory properties, which help to reduce skin redness and irritation [26, 27]. Additionally, the extract of *C. tamariscifolia* exhibited anti‐aging, antioxidant, anti‐wrinkling (inhibition of hyaluronidase), and anti‐inflammatory properties [28, 29]. Collectively, these ingredients work in concert to deliver comprehensive skin benefits, making the formulation highly effective for targeted dermatological applications. Thereby, the tested DC serum offers a comprehensive approach to addressing PIHP.

This study highlights the efficacy of a 2‐MNG‐containing DC serum as a promising intervention for various skin concerns. The serum demonstrated significant improvement in global PAHPI scores, with clinically discernible changes as early as D56, and continuous objective improvement in ITA. These findings underscore the serum's potency in mitigating the multifaceted aspects of acne‐induced PIHP. Additionally, the notable improvements in mean darkness (84% improvement by D84, noticeable by D28) and reduction of Delta *E* (60% reduction at D84) further illustrate the serum's favorable impact on the overall complexion of patients. Our findings also highlight a convergence between dermatologists, patients reported outcomes, and objective instrumental assessments.

Understanding the impact of stigmatization of pigmentary disorders have recently been reported to be very crucial [30]. Research has shown that resolving pigmentary disorders can enhance QOL by reducing self‐consciousness and the feeling of being scrutinized by others [31].

An observational cross‐sectional study aimed to quantify perceived stigmatization among patients with various skin diseases addressing the significant psychosocial burden stigmatization places on these patients [30]. It underscored that factors, including body dysmorphic concerns and distress, were the strongest predictors of stigmatization in pigmentary disorders such as melasma. This also stresses the need for effective treatments for pigmentary disorders due to their prevalence and impact on QOL. In this hereby study, similar stigmatization findings were highlighted at D0 for PIHP whereby the DC serum positively highlighted the improvement in QOL of subjects by D84. These insights have the potential to help clinicians to develop targeted patient management strategies, including evidence‐based interventions [30]. Healthcare providers could consider the effects of PIHP on health‐related QOL and educate patients on available treatment options [31]. This study underlines the psychological impact of PIHP and potential for a DC serum to improve it alongside clinical signs. On top tolerance was excellent in 100% of the subjects which is key when managing PIHP, more prone to worsening in case of irritations.

Although this study establishes a comprehensive approach to addressing post‐acne PIHP, a relatively unexplored area, it has a notable limitation: the small sample size of 32 subjects without a control group. Despite the robustness provided by both clinical and instrumental evaluations, future research would benefit from an increased sample size. Photoprotection is the first line measure when managing hyperpigmentary disorders. Indeed, use of a broad‐spectrum sunscreen with high SPF and strong UVA protection is recommended in populations affected by PIHP to prevent worsening/further darkening of spots [20, 32]. In this study, no additional sunscreen was provided, although patients were advised to protect themselves from the sun. This was to assess the effect of the serum as a stand‐alone treatment. This study demonstrated the efficacy and tolerability of the serum in the treatment of post‐acne PIHP. Objective measurements of ITA and Delta *E* confirmed the positive clinical outcomes. The excellent cosmetic acceptability is likely to enhance adherence in real‐world settings. The reduction in the stigmatization score underscores the impact of PIHP and its improvement over time. These results are highly encouraging, suggesting that the new serum, when used in conjunction with a UVA/visible light filtration sun protection, could further benefit patients.

The aim of this study was to confirm the independent efficacy of a product to address PIHP within a standalone context without introducing other products. Consequently, a split‐face study design which is commonly used for comparative evaluations was not employed. Instead, a more straightforward approach whereby a single‐group design was chosen to ensure that results could be interpreted without the influence of side‐by‐side comparison. However, future studies could be conducted to compare its performance with other products by using a split‐face study.

While this study was oriented toward investigating the potent action of the DC serum as stand‐alone treatment without sunscreen, it would be worthwhile to explore the efficacy of DC serum with regard to other hyperpigmentation disorders such as solar lentigo or melasma. This would provide more insight into different disorders.

## 5 Conclusion

The present study aimed to evaluate the efficacy of a 2‐MNG‐containing dermocosmetic (DC) serum in improving facial PIHP in a post‐acne setting and enhancing the stigmatization of affected patients. The promising results highlight the potential of this novel 2‐MNG‐containing DC serum as a treatment option for PIHP and is a promising candidate for other facial hyperpigmentary disorders, such as melasma and solar lentigos.

## Author Contributions

A.L.D.‐F. was involved in the design, collection, analysis, interpretation, preparation, and decision to submit manuscript for publication. G.P. and D.J. were involved in the conduct, collection, and interpretation. C.L.F. and D.K. were involved in analysis of picture and substantial contribution in revising manuscript. All authors reviewed and approved the manuscript before submission and have agreed to be accountable for their contributions.

## Ethics Statement

The global protocol was submitted and obtained a favorable opinion without restriction by Life Together Ltd‐Ethics Committee (ECC‐COS‐083) and approved on March 8, 2023 in Phoenix, Mauritius.

## Consent

Informed consent was acquired from all patients involved in this study.

## Conflicts of Interest

A.L.D.‐F., G.L.D., D.K., and C.L.F. are all employees of L'Oréal. G.P. and D.J. are employees of CIDP Ltée, the CRO that conducted the study.

## Data Availability

The data that support the findings are available from the corresponding author upon reasonable request.
